# Bed Bugs (Hemiptera: Cimicidae) Population Diversity and First Record of *Cimex hemipterus* in Paris

**DOI:** 10.3390/insects12070578

**Published:** 2021-06-25

**Authors:** Dahlia Chebbah, Nohal Elissa, Denis Sereno, Omar Hamarsheh, Anthony Marteau, Julie Jan, Arezki Izri, Mohammad Akhoundi

**Affiliations:** 1 Parasitology-Mycology Department, Avicenne Hospital, AP-HP, 93009 Bobigny, France; dahlia.chebbah@gmail.com (D.C.); anthony.marteau@aphp.fr (A.M.); arezki.izri@aphp.fr (A.I.); 2 Service Parisien de Santé Environnementale, Sous-Direction de la Santé (SPSE), Mairie de Paris, 75019 Paris, France; nohal.elissa@paris.fr; 3 Unité des Virus Émergents (UVE: Aix-Marseille Univ-IRD 190-Inserm 1207-IHU Méditerranée Infection), 13005 Marseille, France; 4 Institut de Recherche pour le Développement, MIVEGEC, 34032 Montpellier, France; denis.sereno@ird.fr; 5 Institut de Recherche pour le Développement, InterTryp, 34032 Montpellier, France; 6 Department of Biological Sciences, Al-Quds University, Jerusalem 51000, Palestine; ohamarsheh@gmail.com; 7 Agence Régionale de Santé (ARS) Île-de-France, 35, Rue de la Gare, CEDEX 19, 75935 Paris, France; julie.jan@ars.fr

**Keywords:** *Cimex lectularius*, *C. hemipterus*, molecular identification, genetic diversity, sympatry

## Abstract

**Simple Summary:**

The bed bugs, *Cimex lectularius* and *C. hemipterus*, have undergone a significant resurgence worldwide since the 1990s. Despite reports of bed bug infestations being on the rise in recent years in France, little is known about the geographical dispersion, species composition, and inter-and intraspecific genetic variation among bed bug populations in the Paris area. The collection of 1211 samples of bed bugs from different infested locations in Paris (15 arrondissements) and its suburb cities (18 cities) allowed us to highlight *C. lectularius* as the predominant species and to document for the first time the presence of *C. hemipterus* in four infested locations (15th and 19th arrondissements, Bobigny, and Villejuif) in the Paris area. Successful bidirectional sequencing of the cytochrome oxidase 1 (COI) gene for 132 specimens representing processed locations revealed two subpopulations of *C. lectularius* in Paris by neighbor-joining and network phylogenetic analyses. These results provide essential information for further epidemiological and public health studies and may help control management strategies in this metropolis.

**Abstract:**

*Cimex lectularius* and *C. hemipterus* are blood-sucking insects with a long history of presence in human communities. We investigated the molecular diversity of the bed bug population of Paris and its suburb cities using cytochrome oxidase 1 (CO1) sequencing. A total of 1211 specimens belonging to different life stages were collected from 62 infested human dwellings in Paris (13 out of 20 arrondissements) and the surrounding cities (18 cities). Morphological determination and COI sequencing of 132 specimens demonstrated *C. lectularius* as the predominant species and, surprisingly, the presence of *C. hemipterus* in four infested areas of Paris and its suburb cities. Neighbor-joining tree and network analyses depicted the presence of two *C. lectularius* populations. Most samples from Paris and its suburb cities clustered in a major clade. The second population encompasses specimens from Paris (arrondissements 11 and 19) and its suburb cities (e.g., Bobigny, Pantin, and Montreuil) that clustered with Hungary, Czechia, and Finland. This is the second evidence for *C. hemipterus* infestation in France and the third in Europe, which challenges the classic pattern of *C. hemipterus* dispersion and implies sympatric occurrence of *C. lectularius* and *C. hemipterus*. Since Paris is one of the most popular tourist destinations in the world, our observations shed light on bed bugs’ dispersal dynamic and may help future vector control strategies.

## 1. Introduction

Bed bugs (Hemiptera: Cimicidae) are arthropod ectoparasites belonging to the Cimicidae family that feed exclusively on blood. This family comprises more than one hundred species, of which two express a high degree of trophic preference for humans [1]. *Cimex lectularius* is commonly encountered in temperate regions, while *C. hemipterus* is mainly present in tropical and subtropical areas [1,2]. Both of them have a long history of cohabitation with human communities. Over the last two decades, bed bug infestation of human habitats has increased drastically [3]. The infestation occurs in all ethnic groups and at all socioeconomic levels. The resurgence is attributed mainly to an increase in international travel and the development of insecticide-resistant populations [4,5]. Bed bugs are responsible for several clinical and psychological disorders [6,7]. They harbor over 45 infectious agents, including bacteria (such as *Borrelia recurrentis*, *B. duttoni*, *Coxiella burnetii,* and *Rickettsia rickettsii*), fungi (e.g., *Aspergillus* spp.), viruses (e.g., hepatitis B and HIV), filariae, and parasites [6,8]. Despite experimental reports of *C. lectularius*’s ability to transmit pathogens such as *Trypanosoma cruzi* or *Bartonella quintana* in the laboratory [9,10], no evidence showing its role in endemic areas is available [11].

The worldwide resurgence of bed bug infestation has led to a questioning of the underlying factors that govern such geographical expansion. Therefore, additional knowledge on the biology, epidemiology, and genetic structure of bed bugs is required to understand their dispersal patterns at a local or international scale, helping control management strategies against these ectoparasites. Several studies have investigated the dispersal of bed bug species and their population genetics structure in Asia [12], Europe [13], Australia [14], and the USA [15]. In France, despite numerous concerns reported by health centers, private pest control practitioners (PCPs), and municipalities, little is known about the species composition and population genetics of bed bugs. The first study on the genetic diversity of bed bug populations, conducted by Akhoundi et al. [16], revealed a restricted gene flow among south of France bug populations. According to multiple independent reports, bed bug infestation appears to be a public health concern in Paris [17]. Due to the importance of this issue and to fill the knowledge gap, we undertook the present study using morphological and molecular approaches to explore the species composition, genetic diversity, and geographical dispersion of bed bug populations in Paris and its suburb cities.

## 2. Materials and Methods

### 2.1. Bed Bug Collection

Bed bugs were collected within private houses, apartments, building complexes (HLM: Habitation à Loyer Modéré), nursing homes for the elderly (EPHAD: Etablissements d’Hébergement pour Personnes Agées Dépendantes), and immigrant residences in Paris and the surrounding cities. These locations were scrutinized, focusing mainly on the mattresses, bed frames, wall crevices, carpets, and furniture. The notification of bed bug bites, the presence of black fecal spots, and exoskeletons on surfaces were considered the top signs of bed bug infestation. Sampling was carried out with a handheld vacuum cleaner (Dyson V7 trigger, Paris, France) or entomological forceps (Insecta-Pro^®^). Collected specimens were placed in 40 mL sterile mini-glass vials together with a piece of folded bound paper representing an artificial shelter to avoid excessive mortality of bed bugs. Collected bed bugs were brought to the laboratory and identified morphologically under a stereomicroscope (Olympus SZ61, Yokohama, Japan), according to identification keys published by Usinger [18] and Walpole [19]. All the specimens were labeled individually and kept at −20°C for further molecular analysis.

### 2.2. DNA Extraction and PCR Amplification 

Bed bug DNA extraction was carried out individually using Chelex 10% (Bio-Rad, Marnes-la-Coquette, France) [20] and quantified with Qubit (Thermoscientific, Waltham, MA, USA). The extracted specimens’ DNA was subjected to conventional PCR targeting the cytochrome oxidase 1 (COI) gene. Each PCR reaction was performed in a final volume of 25 μL, with 12.5 μL master mixture, 8 μL distilled water, 1 μM of each of the forward (COIF: 5’-GCATTYCCACGAATAAATAAYATAAG-3’) and reverse (COIR: 5’-TAAACTTCTGGATGTCCAAAAATCA-3’) primers and 2.5 μL extracted DNA [12]. The amplification was performed under the following conditions: initial denaturation for 2 min at 95 °C, followed by 5 cycles of 40 s at 94 °C, 40 s at 45 °C, 1 min at 72 °C; and then 35 cycles of 40 s at 94 °C, 40 s at 51 °C, 1 min at 72 °C, and 5 min at 72 °C. A couple of negative and positive controls were used for each PCR batch. The amplicons were analyzed using electrophoresis on 1.5% agarose gel containing ethidium bromide. 

### 2.3. Phylogenetic Reconstruction and Species Assignation

PCR products were subjected to bidirectional DNA sequencing using the same primer pairs used for amplification. The acquired sequences were identified at the species level, based on identity equal to or more than 99% compared with homologous sequences collected in GenBank, using the Basic Local Alignment Search Tool (BLAST) (www.ncbi.nlm.nih.gov/BLAST (accessed on 24 June 2021)). All nucleotide sequences were deposited in GenBank with the assigned accession numbers of XN632157 to XN632269. Sequence alignment was performed with the BioEdit v7.0.0 software [21], and the phylogenetic analysis was carried out using MEGA v.6 software [22]. The inferred phylogenetic tree of *Cimex* species (identified in this study) and homonym sequences from GenBank were constructed using the neighbor-joining (NJ) method and bootstrap values, determined by 1000 replicates. To display the genetic relationships within *Cimex* populations, the median-joining algorithms were implemented using NETWORK v. 5 software [23].

## 3. Results

**Figure 1:**
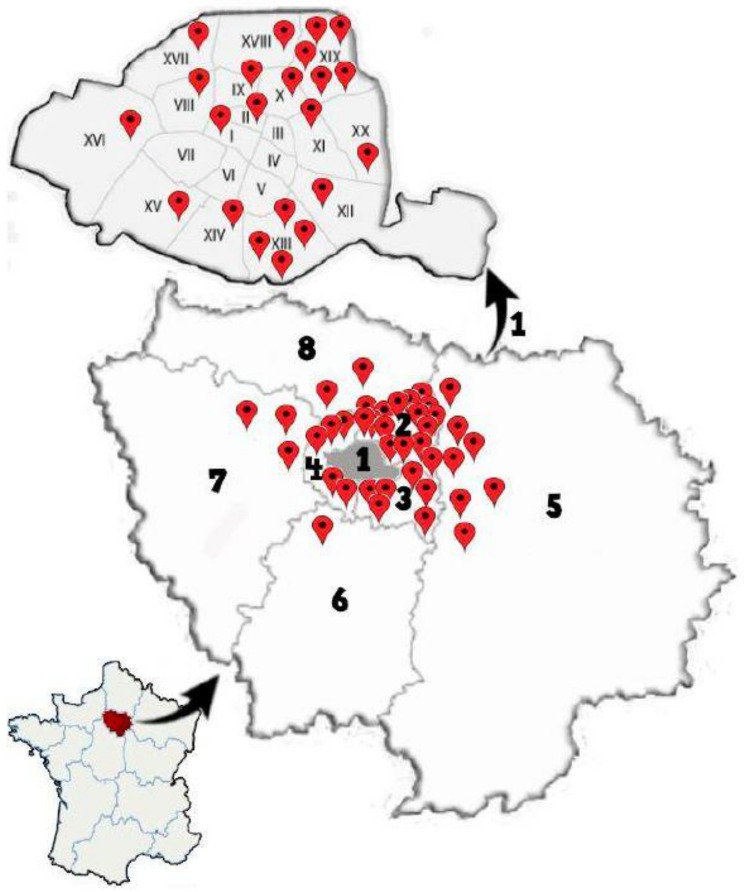
Geographical topology of the processed locations for bed bug sampling in Ile-de-France, France. 1. Paris arrondissements, 2: Seine-Saint-Denis, 3: Val-de-Marne, 4: Hauts-de-Seine, 5: Seine-et-Marne, 6: Essonne, 7: Yvelines, and 8: Val-d’Oise. The arrondissements of Paris are demonstrated by I to XX symbols.

The study was carried out from January to June 2019, in collaboration with the Paris municipality’s public health department (SPSE), who provided the preliminary list of infested locations. A total of 62 human dwellings including 17 private houses, 29 apartments, 12 HLM building complexes, 2 EHPAD, and 2 immigrant residences in 15 arrondissements of Paris (1, 2, 8, 9, 10, 11, 12, 13, 14, 15, 16, 17, 18, 19, and 20) and 18 suburb cities (Arcueil, Asnieres, Aubervilliers, Bobigny, Chilly-Mazarin, Creteil, Drancy, La Courneuve, Marly-le-Roi, Meudon, Montreuil, Nanterre, Neuilly sur Marne, Nogent-sur-Marne, Pantin, Sarcelles, Stains, and Vincennes) were examined for the presence of bed bugs (the sampling details are given in our previously published article [24]). Among them, 56 sites were found to be infested, while in 6 places, no bed bug was collected. A total of 1215 specimens belonging to different life stages (egg, nymph, adult male and female, unfed, and blood-fed) were collected (Figure 1) [24]. According to specific morphological criteria, the adult specimens were identified, which allowed discrimination between *C. hemipterus* and *C. lectularius* [18,19]. Most importantly, the thorax’s first segment (pronotum) expands laterally and presents more flattened extreme margins in *C. lectularius* than *C. hemipterus*. Using these criteria, we recorded *C. lectularius* as the predominant species in the diverse infested locations. Nevertheless, two specimens from the 15th and 19th arrondissements of Paris and two from Bobigny and Villejuif cities were identified as *C. hemipterus*. 

**Figure 2:**
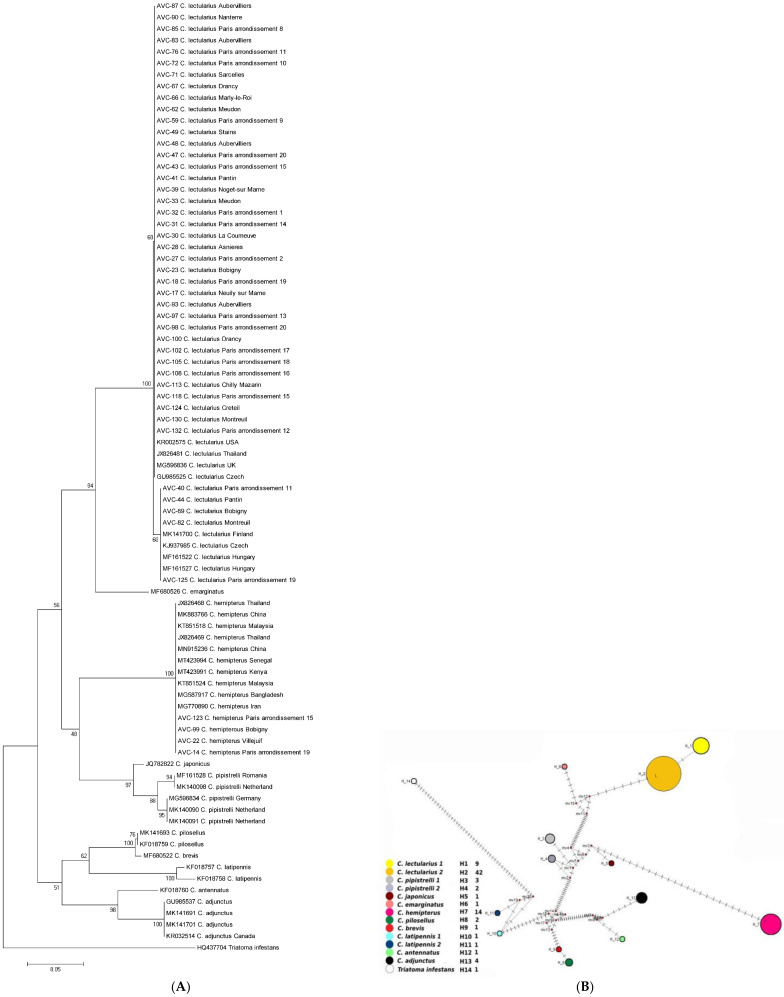
(**A**) Neighbor-joining (NJ) tree reconstructed from COI sequences of bed bug specimens collected in the present study (beginning with AVC) and from sequences collected from GenBank. (**B**) Global analysis median-joining network for various *Cimex* populations. Circle size and circle color indicate frequency and geographical location of haplotypes, respectively. Haplotype numbers are written next to the corresponding circle.

**Figure 3:**
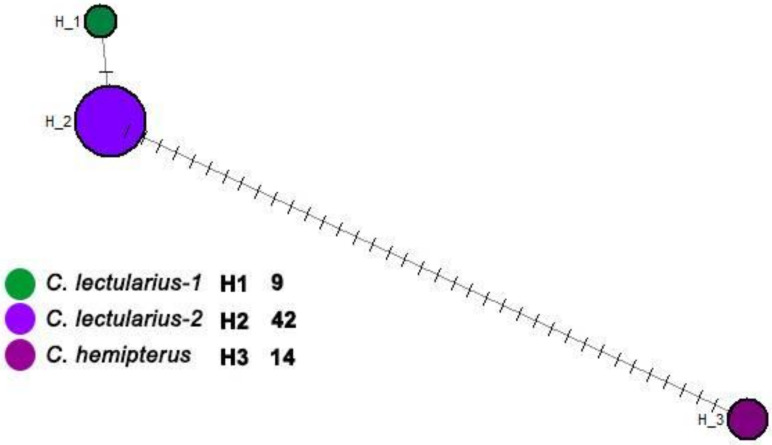
Median-joining network for COI sequences of *C. lectularius* and *C. hemipterous* specimens processed in the present study.

The COI gene fragment was successfully amplified in 132 samples. The 579 base pair fragments’ sequences showed ≤99% identity to *Cimex* sequences from GenBank, which confirmed morphological identification of the *Cimex* species collected in Paris arrondissements and suburb cities. The phylogenetic analysis displayed that the Paris area samples fell within the clade of *C. lectularius* and *C. hemipterus* as human ectoparasitic species. They were divergent from other bat or bird *Cimex* species such as *C. pipistreli*, *C. adjunctus*, and other sequences collected representative of *C. antennatus*, *C. latipennis*, *C. brevis*, *C. pilosellus*, *C. japonicas*, and *C. emaginatus*. This consensus tree revealed two subpopulations within *C. lectularius* samples in the Paris area with bootstrap support values equal to 60% (Figure 2A). Furthermore, the clustering displayed by the median-joining network agrees well with the topology of the phylogenetic trees generated by the neighbor-joining analysis. Some genetic diversities among and between the human-associated species and populations of *Cimex* (*C. lectularius* and *C. hemipterus*), as well as in comparison to the bird/bat-associated *Cimex* species, were observed in the network analysis (Figure 2B). The second median-joining network, including the COI sequences of *C. lectularius* and *C. hemipterous* specimens processed in the present study, indicates a significant genetic differentiation among these populations in areas of Paris (Figure 3). The distribution of COI haplotypes within *Cimex* species in areas of Paris and other countries (registered in the GenBank) and the distribution of COI haplotypes within *C. lectularius* populations processed in the present study are shown in Table 1 and Table 2. Furthermore, an estimated evolutionary divergence between human-associated bed bugs (*C. lectularius* and *C. hemipterus*), and among all *Cimex* species including bird/bat bugs, are given in Files S1 and S2.

## 4. Discussion

France is the most visited country globally, with 89 million tourists reported in 2019. The French capital, Paris, is also Europe’s top tourist destination with a massive draw for foreign visitors—over 30 million tourists, more than any other city in the world [25]. Considering Paris’ touristic importance, the city may also be one of the top destinations for bed bugs via national and international travels. *Cimex lectularius* is the prevalent species reported in France [16,26], but *C. hemipterus* has also been reported in Marseille [27]. The present study is the first to confirm *C. hemipterus* in Paris and the second case documented in France. This species’ presence was reported recently for the first time in Italy [28]. Our case is the third report of *C. hemipterus* infestation in Europe to the best of our knowledge. *Cimex hemipterus* is frequent in tropical regions of Southeast Asia, Africa, and South America [1]. The presence of this tropical species in European countries pinpoints a possible diffusion and colonization of this ectoparasite in these temperate regions. The infestation has probably occurred via passive (e.g., via luggage) transportation by passengers from endemic areas.

*Cimex lectularius* is distributed in Nearctic and Palearctic areas, including Asia, Australia, Africa, and South America [1,29], whereas *C. hemipterus* is common in tropical and subtropical regions [30,31]. Nevertheless, some factors such as international travel, immigration, and secondhand business have disrupted this classic division and extended their geographical dispersions, resulting in the sympatric occurrence of *C. lectularius* and *C. hemipterus*. This would lead to a dramatic increase in bed bug infestations by these species worldwide [1,29]. 

Nowadays, molecular techniques represent a promising approach for diagnosing morphologically close species [32]. Cytochrome oxidase 1 (COI) is a conserved mitochondrial gene showing sufficient genetic variation to be applied in species identification [33]. We demonstrated the mitochondrial COI gene’s effectiveness in discriminating two human parasitic bed bugs. Based on phylogenetic analysis, all sequences were concordantly clustered into the same species group following observations previously reported by Seri Masran et al. [12] and Balvin et al. [13]. The *Cimex* species with a trophic preference for humans (*C. lectularius* and *C. hemipterus*) were clustered separately compared with other bat or bird bugs.

Interestingly, we observed two subpopulations of *C. lectularius*, in which most specimens from diverse geographical areas of Paris and its suburb cities clustered in a major clade. The second subpopulation encompasses the samples from Paris (arrondissements 11 and 19) and its suburb cities (e.g., Bobigny, Pantin, and Montreuil), together with sequences from Hungary (MF161522, 161527), Czechia (KJ937985), and Finland (MK141700) (Figure 2A). These findings were confirmed by network analysis demonstrating significant genetic diversity among *C. lectularius* populations in Paris (Figure 2B and Figure 3).

## 5. Conclusions

This is the first large-scale study investigating the genetic diversity of bed bugs gathered in Paris (13 out of 20 arrondissements) and the surrounding cities (18 cities). Herein, we highlight *C. lectularius* as the predominant species collected and report, for the first time, *C. hemipterus* in Paris (15th and 19th arrondissements) and its suburb cities (Bobigny and Villejuif). Specimens of *C. lectularius* and *C. hemipterus*, processed in the present study, clustered tightly with their counterparts identified in other countries of Europe, Asia, and the USA and were distinct from other bird or bat *Cimex* species (Figure 1). Considering that Paris is one of the most popular tourist destinations in the world, these results provide knowledge on the diversity and infestation pattern of *Cimex* species in Paris. This information is essential in developing bed bug control strategies in this metropolis.

## Figures and Tables

**Table 1:** Distribution of COI haplotypes within *Cimex* species in Paris, France and other countries.

*Cimex* Species	COI Haplotypes		^1^ Regional Distribution
	Haplotype	Frequency	
*C. lectularius*	H01	9	Paris (5), Finland, Czechia, Hungary (2)
	H02	42	Paris (39), USA, UK, Czechia
*C. pipistrelli*	H03, H04	5	Germany, Netherlands (3), Romania
*C. japonicus*	H05	1	
*C. emarginatus*	H06	1	
*C. hemipterus*	H07	14	Paris (4), China (2), Thailand (2), Malaysia (2), Iran, Bangladesh, Senegal, Kenya
*C. pilosellus*	H08	2	
*C. brevis*	H09	1	
*C. latipennis*	H10, H11	2	
*C. antennatus*	H12	1	
*C. adjunctus*	H13	4	

^1^ Numbers in brackets indicate the haplotype numbers in each region.

**Table 2:** Distribution of COI haplotypes within *C. lectularius* populations collected from the Paris area, France.

Areas of Paris	Haplotypes	Frequency
Paris arrondissements	H1, H2	2, 18
Meudon	H2	2
Neuily sur Marne	H2	1
Asnieres	H2	1
La Courneuve	H2	1
Pantin	H1, H2	1, 1
Aubervilliers	H2	4
Stains	H2	1
Marly le Roi	H2	1
Drancy	H2	2
Sarcelles	H2	1
Noget sur Marne	H2	1
Nanterre	H2	1
Chilly Mazarin	H2	1
Creteil	H2	1
Montreuil	H1, H2	1, 1
Bobigny	H1	1

## Data Availability

Not applicable.

## Supplementary Materials

The following are available online at https://www.mdpi.com/article/10.3390/insects12070578/s1, File S1: Estimates of Evolutionary Divergence between human associated bed bug (*C. lectularius*, *C. hemipterus*) sequences comparing to bird/bat associated Sequences, File S2: Estimates of Evolutionary Divergence among human associated bed bug (*C. lectularius*, *C. hemipterus*) sequences.
